# Internalization of AMPA-type Glutamate Receptor in the MIN6 Pancreatic β-cell Line

**DOI:** 10.1247/csf.20020

**Published:** 2020-06-24

**Authors:** The Mon La, Hiroshi Yamada, Sayaka Seiriki, Shun-AI Li, Kenshiro Fujise, Natsuho Katsumi, Tadashi Abe, Masami Watanabe, Kohji Takei

**Affiliations:** 1 Department of Neuroscience, Okayama University Graduate School of Medicine, Dentistry and Pharmaceutical Sciences, Okayama 700-8558, Japan; 2 Center for Innovative Clinical Medicine, Okayama University Hospital, Okayama 700-8558, Japan

**Keywords:** endocytosis, GluR2, AMPA, cortactin, MIN6

## Abstract

The activity of AMPA-type glutamate receptor is involved in insulin release from pancreatic β-cells. However, the mechanism and dynamics that underlie AMPA receptor-mediated insulin release in β-cells is largely unknown. Here, we show that AMPA induces internalization of glutamate receptor 2/3 (GluR2/3), AMPA receptor subtype, in the mouse β-cell line MIN6. Immunofluorescence experiments showed that GluR2/3 appeared as fine dots that were distributed throughout MIN6 cells. Intracellular GluR2/3 co-localized with AP2 and clathrin, markers for clathrin-coated pits and vesicles. Immunoelectron microscopy revealed that GluR2/3 was also localized at plasma membrane. Surface biotinylation and immunofluorescence measurements showed that addition of AMPA caused an approximate 1.8-fold increase in GluR2/3 internalization under low-glucose conditions. Furthermore, internalized GluR2 largely co-localized with EEA1, an early endosome marker. In addition, GluR2/3 co-immunoprecipitated with cortactin, a F-actin binding protein. Depletion of cortactin by RNAi in MIN6 cells altered the intracellular distribution of GluR2/3, suggesting that cortactin is involved in internalization of GluR2/3 in MIN6 cells. Taken together, our results suggest that pancreatic β-cells adjust the amount of AMPA-type GluR2/3 on the cell surface to regulate the receptive capability of the cell for glutamate.

## Introduction

The α-amino-3-hydroxy-5-methyl-4-isoxazolepropionic acid (AMPA) receptor is an ion channel-coupled receptor for glutamate that mediates fast synaptic transmission in the central nervous system. Each AMPA receptor was made up of GluR1, GluR2, GluR3 and GluR4 (Diering and Huganir, 2018).

In neurons, AMPA receptor is internalized in response to N-methyl-D-aspartate (NMDA) receptor activation (Beattie *et al.*, 2000) and AMPA binding (Carroll *et al.*, 1999; Lin *et al.*, 2000), and the internalization occurs in a dynamin- and clathrin-dependent manner (Carroll *et al.*, 1999; Man *et al.*, 2000). Following internalization, AMPA receptors are quickly recycled to the neuronal surface under basal or AMPA-stimulated conditions (Lee *et al.*, 2004), or partially delivered to degradation pathways upon NMDA receptor activation (Lee *et al.*, 2004). The resulting decrease of AMPA receptors in number on the cell surface could lower the capacity for glutamate recognition in the synapse. In neuronal cells, cortactin, a F-actin binding protein, directly associates with GluR2 as revealed by proteomics and biochemical studies (Klemmer *et al.*, 2009; Parkinson *et al.*, 2018), and is the interaction implicated in endosomal–lysosomal sorting of the AMPA receptor (Parkinson *et al.*, 2018).

The AMPA receptor is expressed in pancreatic α-, β-, and δ-cells, and is implicated in hormone secretion (Takahashi *et al.*, 2019). By RT-PCR or pharmacological analyses, all AMPA receptor subtypes (GluR1, GluR2, GluR3, and GluR4) are expressed in mouse primary β-cells (Wu *et al.*, 2012) and in the mouse β-cell line MIN6 (Morlry *et al.*, 2000). In addition, AMPA receptor activation stimulates insulin secretion in rat islets (Inagaki *et al.*, 1995), perfused pancreas (Bertrand *et al.*, 1992), and MIN6 cells (Gonoi *et al.*, 1994). However, the mechanism that regulates AMPA receptor-mediated insulin secretion from β-cells is largely unknown.

In this study, we investigated the internalization of GluR2/3 in MIN6 cells, a pancreatic β-cell line. We showed that AMPA stimulated rapid internalization of GluR2/3 in MIN6 cells. In addition, localization of GluR2/3 at the cell surface was partly regulated by cortactin. Our study proposes that glutamate signaling in β-cells is regulated by a similar mechanism as glutamate signaling in neurons.

## Materials and Methods

### Antibodies and reagents

A rabbit polyclonal antibody recognizing the cytoplasmic domain (KQNFATYKEGYNVYGIESVKI) of GluR2/3 (cat# 07-598), a mouse monoclonal antibody recognizing amino acid residues 175–430 of mouse of GluR2 (cat# MAB397), and a mouse monoclonal antibody against cortactin (cat# 05-180 clone 4F11) were purchased from EMD Millipore (Billerica, MA, USA). Goat polyclonal anti-GluR3 antibody (cat# sc7613) was purchased from Santa Cruz Biotechnology Inc. (Santa Cruz, CA, USA). A mouse monoclonal antibody against β-actin (cat# A5441 clone AC-15) was purchased from Merck KGaA (Darmstadt, Germany). A rabbit polyclonal antibody against EEA1 (cat# 3288 C45B10) was purchased from Cell Signaling Technology (Danvers, MA, USA). A mouse monoclonal antibody against α-adaptin (AP2) (cat# CP46) was purchased from Merck KGaA. A mouse monoclonal anti-clathrin heavy chain antibody (cat# MA1-065), Alexa Fluor 488-conjugated anti-mouse immunoglobulin G (IgG; cat# A11001), Rhodamine Red X-conjugated anti-rabbit IgG (cat# R6394), horseradish peroxidase-conjugated goat anti-rabbit IgG (H+L; cat# 31460), and rabbit anti-mouse IgG (H+L; cat# 31450) were purchased from Thermo Fisher Scientific, Inc. (Waltham, MA, USA).

### Cell culture

MIN6 cells were kindly gifted by Dr. Seino (Kobe University), and were cultured in Dulbecco’s Modified Eagle’s Medium (DMEM) (Life Technologies) containing 10% fetal bovine serum (FBS) at 37°C in 5% CO_2_ (Minami *et al.*, 2000).

### Expression and purification of cortactin and its mutants

cDNA encoding full-length rat cortactin or cortactin deletion mutants (1–80 aa, 284–450 aa, and 451–509 aa) were prepared by PCR and subcloned into the pGEX-6P vector as a BamHI/EcoRI fragment (Yamada *et al.*, 2013). The nucleotide sequences of the constructs were verified by DNA sequencing. Glutathione-S-transferase (GST)-tagged proteins were expressed in *Escherichia coli* and purified as described previously (Yamada *et al.*, 2013).

### siRNA-mediated interference

Pre-annealed siRNAs for mouse cortactin and the negative control siRNA were synthesized and purified (Thermo Fisher Scientific). The sequences for the siRNAs for mouse cortactin were as follows: GAAUCCCAAAAAGACUAUAtt-3' (sense), 5'-UAUAGUCUUUUUGGGAUUCat-3' (antisense) for oligo 1; GCAAAUAUGGGAUUGACAAtt-3' (sense), UUGUCAAUCCCAUAUUUGCca-3' (antisense) for oligo 2. A scrambled siRNA with no significant sequence homology to all mouse, rat, or human gene sequences was used as the negative control. The day before transfection, cells were plated in 6-well plates (1×10^6^ cells/well). Cells were transfected with 100 pmol of duplex siRNAs using 10 μl of Lipofectamine RNAi MAX (Thermo Fisher Scientific). After 72 h, cells were used for double-immunofluorescence experiments, as described below.

### Fluorescence microscopy

MIN6 cells were fixed with 4% paraformaldehyde and stained for immunofluorescence as described previously (Yamada *et al.*, 2013). Samples were examined using a spinning disc confocal microscope system (X-Light confocal imager, CrestOptics S.P.A., Rome, Italy) combined with an inverted microscope (IX-71, Olympus Optical Co., Ltd., Tokyo, Japan) and an iXon+ camera (Oxford Instruments, Oxfordshire, UK). The confocal system was controlled by MetaMorph Software (Molecular Devices, Sunnyvale, CA, USA). When necessary, images were processed using Adobe Photoshop CS3 or Illustrator CS3 software.

### Pull-down assay

The GST pull-down assay was performed as described previously (Yamada *et al.*, 2013). Mouse brain was homogenized in buffer A (0.35 M sucrose, 5 mM HEPES-NaOH, 5 μg/ml leupeptin, 5 μg/ml pepstatin A, 5 μg/ml chymostatin, pH 7.4). After removing the nuclear fraction by centrifugation at 1,000 g for 10 min, the supernatant was further centrifuged at 16,000 g for 30 min at 4°C. The resultant pellet was incubated at 4°C for 1 h in lysis buffer (22.5 mM HEPES-NaOH, 150 mM NaCl, 1% Triton X-100, pH 7.4) containing a protease inhibitor cocktail (cat# 11697498001 Roche Diagnostics, Basel, Switzerland). The mixture was then centrifuged at 16,000 g for 30 min at 4°C. The resultant supernatant was used as mouse brain extract. GST-fusion proteins of cortactin (400 μg) bound to glutathione-Sepharose beads (cat# 17-0756-01, GE Healthcare UK Ltd., Buckinghamshire, UK) were incubated with 2.5 mg of mouse brain extract in lysis buffer with a protease inhibitor cocktail at 4°C for 1 h. Bead-bound GluR2 was separated by centrifugation and analyzed by Western blotting using an anti-intracellular GluR2 antibody. For pull-down experiment using MIN6 cell lysate, cells (5×10^6^ cells) were lysed with the buffer (150 mM NaCl, 10 mM Tris-HCl, 0.5 mM EDTA, 10 mM NaF, 1% Triton X-100, pH 7.4) at 4°C. The lysate was centrifuged at 13,000 g for 30 min at 4°C, and the supernatant was used for GST pull-down assay in the manner as to using brain extract.

### Immunoprecipitation

MIN6 cells (5×10^6^ cells) were lysed in buffer A with 1% Triton X-100, 1 mM EDTA, pH 7.9, and a protease inhibitor cocktail (Roche Diagnostics). The lysate was centrifuged at 13,000 g for 30 min at 4°C, and the supernatant was immunoprecipitated. The protein complexes were immunoprecipitated with protein G (cat# 17-0618-01, GE Healthcare UK Ltd., Buckinghamshire, UK) from 2.5 mg of cell extract using 5 μg of the polyclonal anti-intracellular GluR2/3 antibody or pre-immune IgG, and then visualized by Western blotting using a monoclonal anti-cortactin antibody.

### Morphometric analysis

To assess co-localization of GluR2/3 with AP2, clathrin, or cortactin, immunostained cells were photographed, and the immunoreactivity along a randomly selected area of the cell was measured using MetaMorph Software. The line-scanning of fluorescence images was performed using MetaMorph Software.

### Transmission electron microscopy

For immunoelectron microscopy, MIN6 cells were fixed with 4% paraformaldehyde and 0.1% glutaraldehyde in 0.1 M sodium phosphate buffer (PB) (pH 7.4). The fixed cells were immersed in 2.3 M sucrose, 50% polyvinylpyrrolidone in 0.1 M PB (pH 7.4) for several hours, and then rapidly frozen in a Reichert KF-80 Universal Cryofixation System (Leica Microsystems Heidelberg GmbH, Heidelberg, Germany). Frozen ultra-thin sections (60–70 nm thickness) were cut using Reichert Ultracut S ultramicrotome (Leica Microsystems Heidelberg GmbH), and picked up on formvar- and carbon-coated Ni grids (Nissin EM Co., Ltd., Tokyo). For immunogold staining, the sections were washed with 0.1 M PB containing 0.1 M glycine and 1% bovine serum albumin (BSA), blocked with 1% BSA in 0.1 M PB for 10 min, and then incubated with an anti-intracellular GluR2 antibody (1:200, cat# 07-598, END Millipore) for 90 min at room temperature. Specimens were rinsed with 0.1% BSA-PB for 15 min, incubated for 30 min with 10 nm gold-conjugated goat anti-rabbit IgG (1:50, cat# EMGAR10, BBI solutions, Cardiff, UK), washed in 0.1% BSA-PB and 0.1 M PB, and fixed with 2% glutaraldehyde in 0.1 M PB for 10 min. After washing with 0.1 M PB and distilled water, the sections were further fixed with 2% OsO_4_ for 20 min, washed in distilled water, stained with 2% uranium acetate for 20 min, and then coated with 2.2% polyvinyl alcohol. The sections were visualized with an Hitachi H-7100 transmission electron microscope (Hitachi Co., Ltd., Tokyo, Japan).

### Surface labeling assay

A surface biotinylation assay was carried out as previously described (Lin *et al.*, 2000). GluR2 biotinylation was performed at 4°C. MIN6 cells (4×10^6^ cells per well in a 6 well plate) were incubated in low-glucose Ringer’s solution comprising 128 mM NaCl, 1.9 mM KCl, 1.2 mM KH_2_PO_4_, 2.4 mM CaCl_2_, 1.3 mM MgSO_4_, 26 mM NaHCO_3_, 10 mM HEPES-NaOH, 3.3 mM glucose, and 0.2% BSA (pH 7.4). MIN6 cells were labeled for 20 min with EZ-link Sulfo-NHS-SS-biotin (1.5 mg/ml, cat#21331, Thermo Fisher Scientific) in PBS containing 0.1 mM CaCl_2_ and 1 mM MgCl_2_ (PBS (+)) to biotinylate surface proteins. After washing with PBS (+), cells were incubated in low-glucose Ringer’s solution with or without 0.5 mM AMPA (cat#A9111, Merck KGaA) for 15 min at 37°C. Receptor internalization was stopped by rapid cooling to 4°C. Biotinylated proteins remaining on the cell surface were stripped of biotin by the non-permeant reducing agent glutathione (50 mM glutathione, 25 mM NaCl, 10 mM EDTA, 0.2% BSA, and 50 mM Na-phosphate, pH 8.0). Glutathione was subsequently neutralized by 50 mM iodoacetamide in PBS (+). Cells were lysed in lysis buffer (50 mM Tris-HCl, pH 7.4, 2 mM EDTA, 2 mM EGTA, 100 mM NaCl, and 0.2% SDS), sonicated, and boiled for 5 min. After centrifugation at 16,000 g, each supernatant contained an equal amount of total protein was incubated with MagnaBind streptavidin beads (cat#21344, Thermo Fisher Scientific) to capture biotinylated proteins. After washing in lysis buffer, biotinylated proteins were eluted from streptavidin beads by boiling in sample buffer, separated by SDS-PAGE and analyzed by Western blotting using an antibody against intracellular GluR2/3 (1:1000).

To assess GluR2 internalization morphologically, MIN6 cells cultured on coverslips (4x10^6^ cells per well on a 6-well plate) were labeled with anti-extracellular GluR2 antibody (40 μg/ml) in low-glucose Ringer’s solution for 1 h at 4°C. Cells were then washed with ice-cold low-glucose Ringer’s solution five times to remove excessive antibody. Cells were stimulated with 1 mM AMPA in low-glucose Ringer’s solution at 37°C for 20 min. After washing with ice-cold PBS (+), cells were fixed with 4% paraformaldehyde in PBS (+) and were examined by double immunofluorescence.

### Preparation for membrane fraction cell or brain

All experiments and protocols were approved by the Institutional Animal Care and Use Committee of Okayama University (OKU-2019688, Japan). Six-week-old male mice (Shimizu Laboratory Supplies Co., Kyoto, Japan) were anesthetized using sevoflurane, and all efforts were made to minimize animal suffering. Following dissection, whole mouse brain or MIN6 cells were homogenized in PBS containing a protease inhibitor cocktail (Roche Diagnostics) with a Potter-type glass-Teflon homogenizer. The homogenate was centrifuged at 20,000 g for 30 min at 4°C. The supernatant was sampled in SDS sample buffer. Samples were boiled for 5 min and subjected to Western blot analysis.

### Western blotting

Samples were subjected to SDS-PAGE in 10% polyacrylamide gel and transferred electrophoretically to a nitrocellulose membrane (cat# 10600003, GE Healthcare Life Sciences). The membrane was blocked with 140 mM NaCl, 1 mM EDTA, 20 mM Tris-HCl (pH 7.4), containing 0.1% Tween 20 and 5% skimmed milk for 4 h at room temperature, and incubated with primary antibodies (1:1000) for 2 h followed by incubation with peroxidase-conjugated secondary antibodies (1:10000) for 1 h. Bands were visualized by enhanced chemiluminescence Western blotting detection reagents (cat# RPN2106, GE Healthcare Life Sciences). Protein concentration was determined using a bicinchoninic acid assay kit (cat# 23235, Thermo Fisher Scientific, Inc.) with BSA used as the standard.

### Statistical analysis

Statistical analyses were performed using KaleidaGraph software for Macintosh, version 4.1 (Synergy Software Inc., Essex Junction, VT, USA). An analysis of variance and Tukey’s honest significant difference post-hoc test were used to compare several groups. The Student’s t-test was used to compare two groups. P values of less than 0.05 (*) or 0.01 (**) were considered significant.

## Results

### AMPA receptor is expressed intracellularly and at the plasma membrane of MIN6 cells

**Fig. 1:**
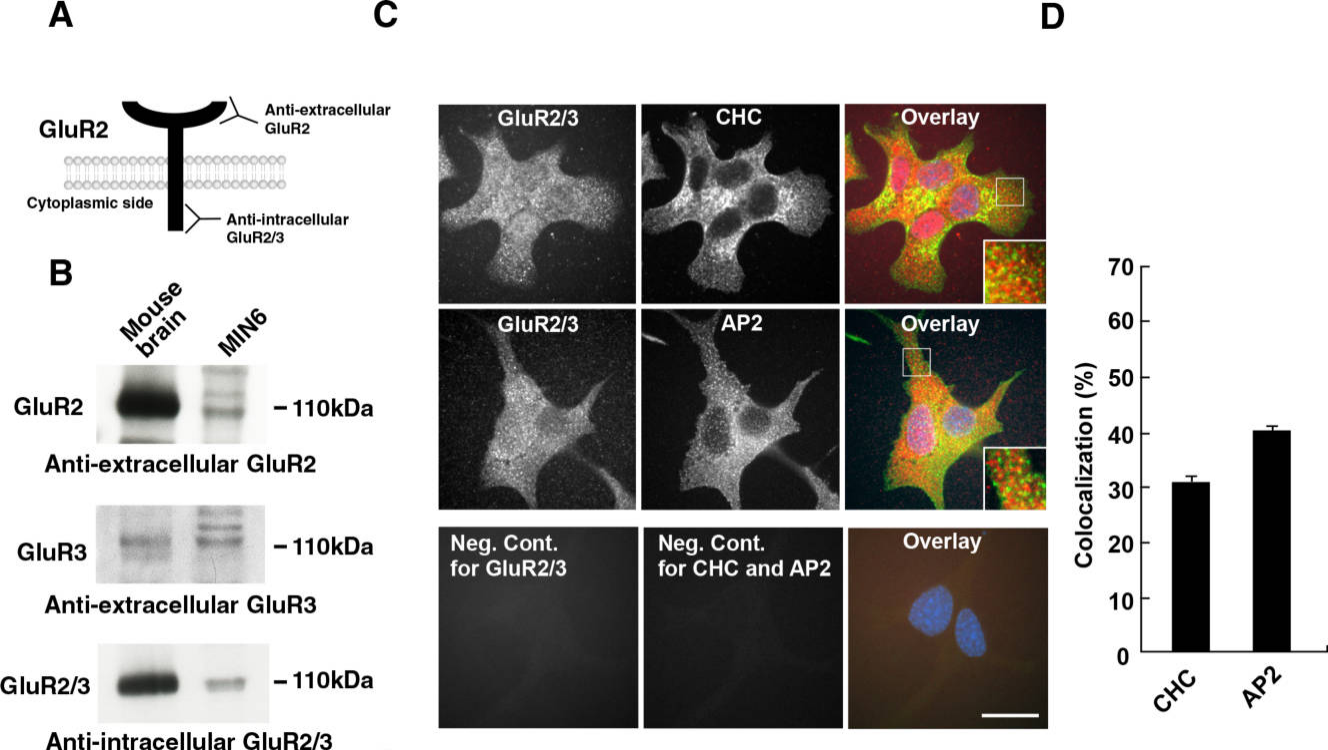
GluR2 and GluR3 is expressed in MIN6 cells. (A) Recognition site for anti-extracellular or -intracellular GluR2 or GluR3 antibody used in the study. (B) Western blotting analyses using anti-extracellular GluR2 (upper panel) or GluR3 (middle panel) or anti-intracellular GluR2/3 (bottom panel) antibodies. Membrane fractions from mouse brain (mouse brain, 10 μg) or MIN6 cell membranes (MIN6, 80 μg) were analyzed. (C) Co-localization of GluR2/3 (labeled using anti-intracellular GluR2/3) with clathrin heavy chain (CHC, upper panel) or AP2 (middle panel) in MIN6 cells. An immunofluorescence image without primary antibody is shown as a negative control (bottom panels). Scale bar, 20 μm. (D) Co-localization measurement of GluR2/3 and CHC or AP2 in MIN6 cells. The data shown are the mean±S.E.M. from 20 cells in three independent experiments. In each sample, co-localization was determined in three randomly selected areas per cell (21 μm^2^) using overlay images.

**Fig. 2:**
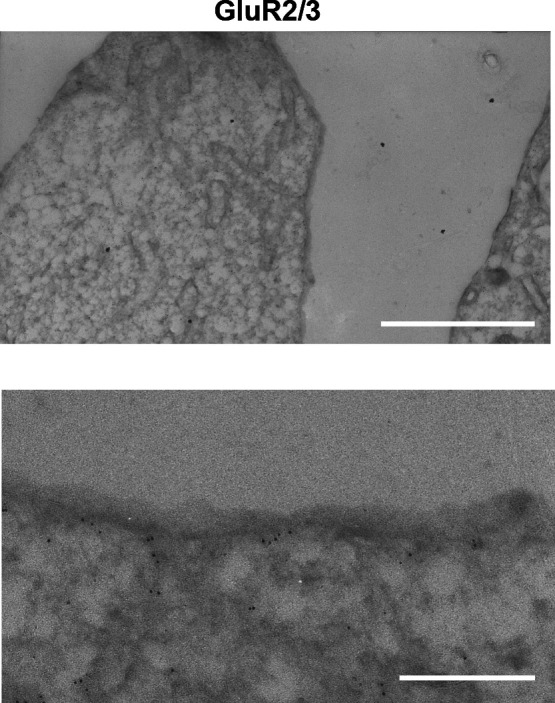
GluR2/3 is localized at the plasma membrane of MIN6 cells. Immunogold electron micrograph of MIN6 cells stained with anti-intracellular GluR2/3 antibodies. Scale bar, 1.5 μm in upper panel; 300 nm in bottom panel.

Previous reports have used pharmacological approaches or measurement of mRNA levels to indicate expression of AMPA receptor, GluR2 and GluR3, in MIN6 cells (Gonoi *et al.*, 1994; Morlry *et al.*, 2000). However, expression of AMPA receptor protein has not been determined. We used site-specific antibodies against extracellular portion of GluR2 or GluR3 to detect these proteins in MIN6 cells by Western blotting (Fig. 1A). The GluR2 and GluR3 proteins were clearly detected in membrane fractions from mouse brain and MIN6 cells (Fig. 1B). In addition, anti-intracellular GluR2/3 antibody, which recognizes both intracellular portion of GluR2 and GluR3 (Lee *et al.*, 2002), also detected the proteins in mouse brain and MIN6 cells. Next, we examined localization of the GluRs in MIN6 cells cultured in DMEM containing high glucose (25 mM). Immunofluorescence using anti-intracellular GluR2/3 antibody showed that the GluR2/3 was visible as dots and observed throughout cells (Fig. 1C). Double-immunofluorescence studies showed that about 30–40% of total intracellular GluR2 co-localized with clathrin heavy chain (CHC) or AP2, markers for clathrin-coated pits and vesicles (Fig. 1D). We further confirmed the subcellular localization of GluR2/3 on the plasma membrane in MIN6 cells by immunoelectron microscopy. In cells stained with an anti-intracellular GluR2/3 antibody, immuno-gold particles were often found on the cytoplasmic surface of the plasma membrane and the internal vacuolar membrane (Fig. 2). These results suggest that GluR2/3 are, at least in part, internalized in MIN6 cells.

### AMPA receptor is internalized by stimulation with AMPA in MIN6 cells

**Fig. 3:**
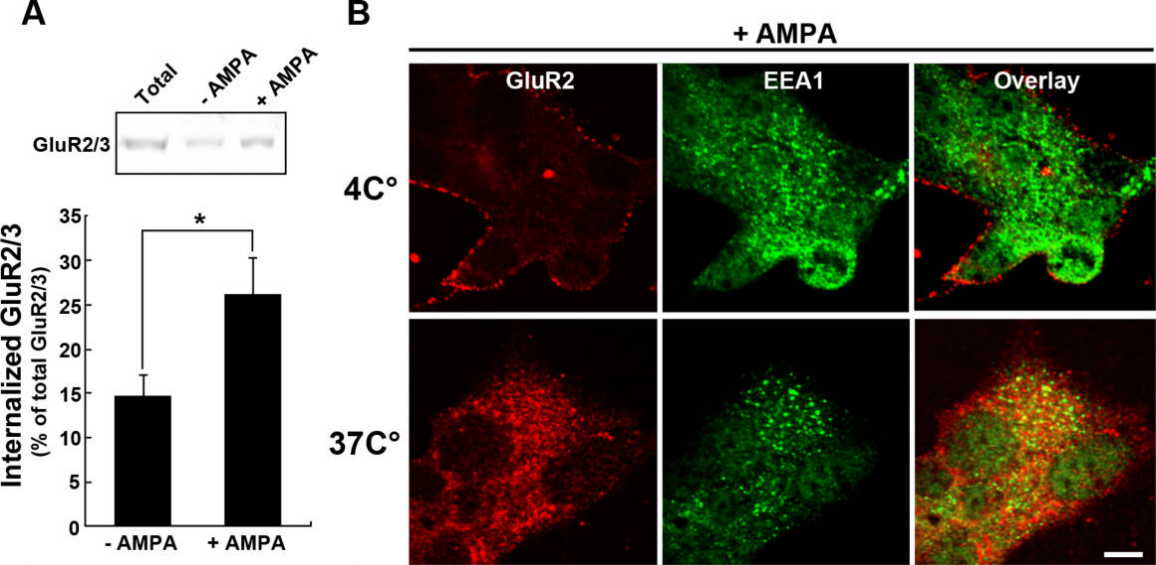
GluR2/3 are internalized upon AMPA stimulation in MIN6 cells. (A) Detection of GluR2/3 internalization by surface biotinylation assay. MIN6 cell surface proteins including GluR2/3 remaining on the plasma membrane were labeled with sulfo-NHS-SS-biotin. After stimulation with AMPA, biotin-labeled receptors were detected by Western blotting (upper panel). Internalized GluR2/3 is shown as a percentage of the total amount of surface-labeled GluR2/3 by densitometric analysis (bottom panel). All results represent mean±S.E.M. from three independent experiments.*, P<0.05 (B) Immunofluorescence detection of internalized GluR2 in MIN6 cells. The extracellular domain of GluR2 was labeled with anti-extracellular GluR2 at 4°C. MIN6 cells were stimulated with AMPA at 4°C (upper panel) or 37°C (bottom panel), and then fixed and stained. Antibody-labeled GluR2 was visualized by double immunofluorescence. Scale bar, 20 μm.

Glutamate are released from pancreatic α-cells under low glucose conditions (Yamada *et al.*, 2001; Hayashi *et al.*, 2003), it is possible that β-cells could respond to the transmitter under the conditions. Next, we examined whether GluR2/3 is internalized in MIN6 cells by ligand-binding under low glucose conditions using two approaches. First, GluR2/3 on the cell surface was labeled with sulfo-NHS-SS-biotin in ice-cold Ringer’s solution containing low glucose. GluR2/3 internalization was initiated by incubating the cells at 37°C, with or without AMPA, to activate intracellular membrane trafficking, and the amount of internalized GluR2/3 was determined biochemically as described previously (Lin *et al.*, 2000). To remove the receptors remaining at the plasma membrane, biotinylated GluR2/3 was cleaved by treatment with glutathione. In the absence of AMPA, an internalization was observed (14.8±2.1% of total biotinylated GluR2/3, n=3). When cells were stimulated with AMPA at 0.5 mM, internalization increased by approximately 1.8-fold compared with internalization without AMPA (26.3±4% of total biotinylated GluR2/3, n=3), suggesting that the GluR2/3 internalization is enhanced by ligand-binding (Fig. 3A).

As an alternative method to detect GluR2 internalization, GluR2 was morphologically traced in MIN6 cells. GluR2 on the cell surface was labeled with anti-extracellular GluR2 at 4°C under low glucose conditions. Membrane trafficking of the receptor was initiated by incubation at 37°C, and localization of antibody-labeled GluR2 was detected by double immunofluorescence. As a negative control, AMPA-stimulated cells were incubated at 4°C; in these cells, antibody-labeled GluR2 remained at the membrane surface (Fig. 3B). At 37°C, GluR2 was internalized (Fig. 3B), and the internalized GluR2 largely co-localized with EEA1, a marker for early endosomes. These results suggest that GluR2 is transported to early endosomes and passes through a recycling pathway.

### Cortactin is involved in the maintenance of intracellular localization of AMPA receptor in MIN6 cells

**Fig. 4:**
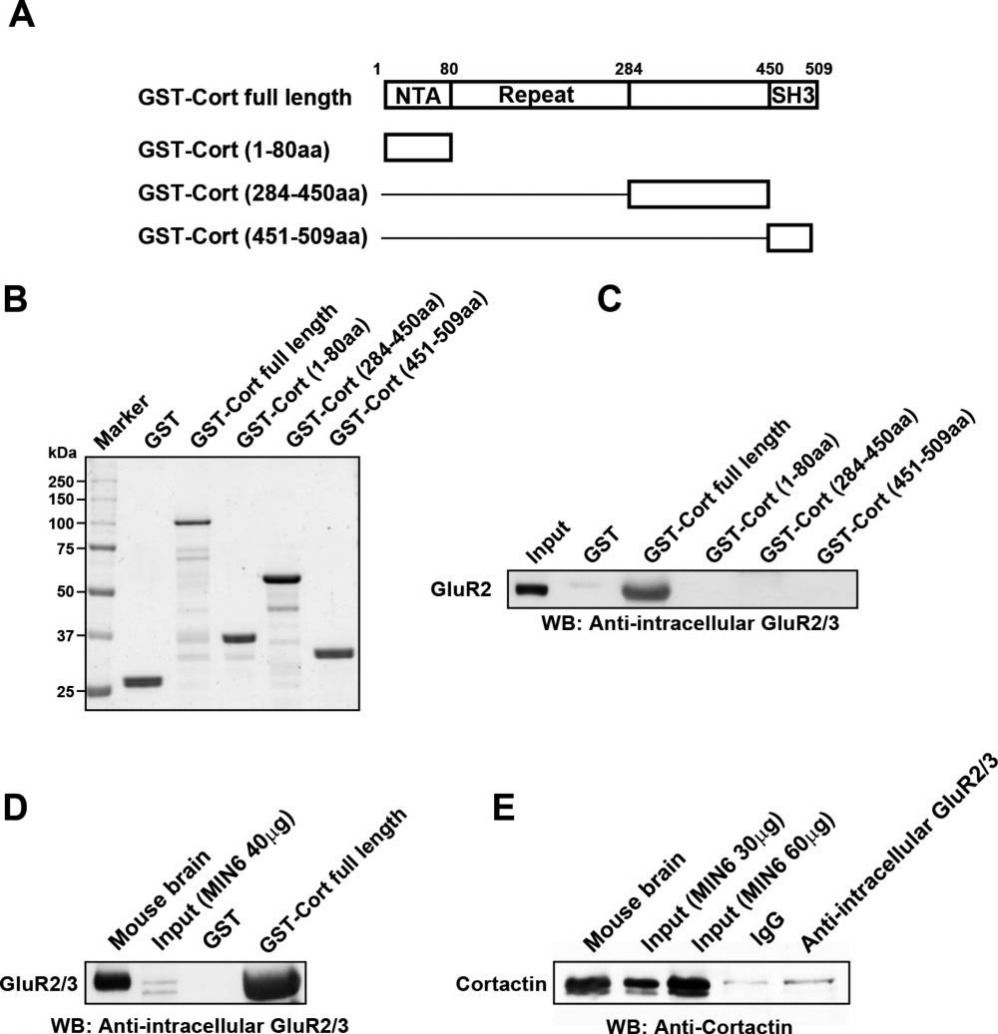
GluR2/3 directly interacts with cortactin in MIN6 cells. (A) Domain structures of the cortactin constructs used in the results presented in panels (B) and (C). NTA, N-terminal acidic region; Repeat, six tandem repeat region; SH3, Src homology 3. (B) SDS-PAGE of GST-fusion proteins used in the results presented in panel (C). Five micrograms of the indicated purified GST-fused cortactin mutants were analyzed. (C) Four-hundred micrograms of full-length GST-cortactin (GST-Cort full length), GST-cortactin (1–80 aa) (GST-Cort (1–80 aa)), GST-cortactin (284–450 aa) (GST-Cort (284–450 aa)), or GST-cortactin (451–509 aa) (GST-Cort (451–509 aa)) bound to glutathion beads were incubated with 2.5 mg of whole mouse brain extract and 1% Triton X-100 for 1 h. GluR2/3-bound to the beads was analyzed by Western blotting. Whole mouse brain extract (20 μg) was used as a control (input). (D) Four-hundred micrograms of full-length GST-cortactin (GST-Cort full length) bound to glutathion beads were incubated with 2.5 mg of MIN6 cell lysate for 1 h. GluR2/3-bound to the beads was analyzed by Western blotting. Whole MIN6 cell lysate (40 μg) was used as a control (input). (E) Immunoprecipitation demonstrating an in vivo interaction between GluR2/3 and cortactin. MIN6 cells were solubilized with 1% Triton X-100. The protein complexes were immunoprecipitated using anti-intracellular GluR2/3 antibodies or pre-immune IgG, and then visualized by Western blotting with a monoclonal anti-cortactin antibody. Total cell lysates (input; 30 or 60 μg) were used as a control.

Recently, GluR2 was shown to directly interact with cortactin via its repeat domain, and this interaction is implicated in endosomal–lysosomal sorting in neuronal cells (Parkinson *et al.*, 2018). Therefore, we next confirmed the interaction between cortactin and GluR2 in mouse brain extract with pull-down assay using four kinds of recombinant cortactin proteins (Fig. 4A, B). Consistent with the previous report (Parkinson *et al.*, 2018), anti-intracellular GluR2/3 antibody clearly detected 110 kDa protein from the precipitate with GST-tagged full-length cortactin from mouse brain extract (Fig. 4C). The repeat domain-lacking mutants, cortactin (GST-Cort (1–80 aa)), cortactin (GST-Cort (284–450 aa)), or cortactin (GST-Cort (451–509 aa)) did not precipitate GluR2 (Fig. 4C). Furthermore, GST-tagged full-length cortactin precipitated the receptors from MIN6 cell lysates (Fig. 4D). Association of cortactin and GluR2/3 in MIN6 cells was also examined by co-immunoprecipitation. GluR2/3 protein labeled with an anti-intracellular GluR2/3 antibody co-immunoprecipitated with cortactin in MIN6 cell lysates (Fig. 4E).

**Fig. 5:**
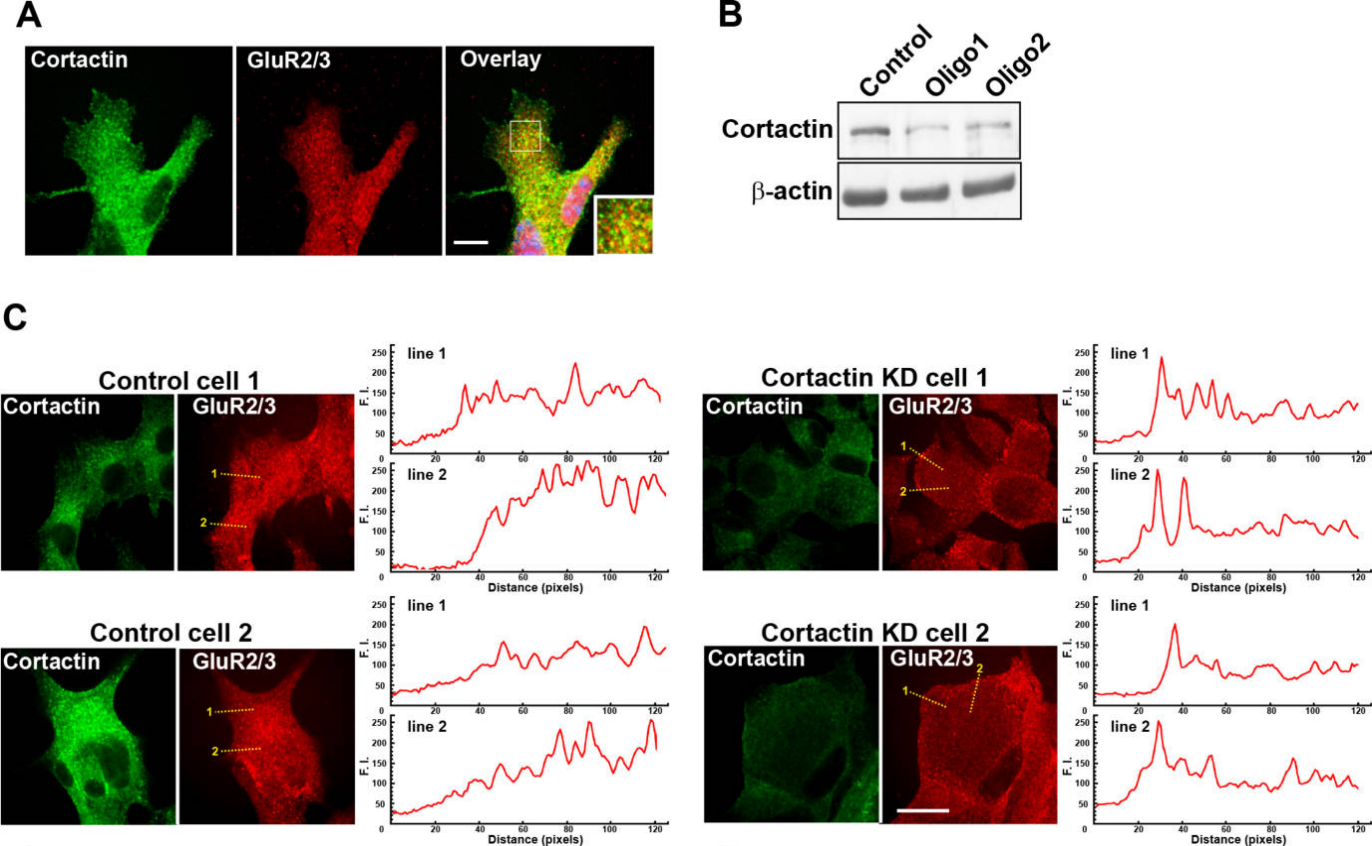
Depletion of cortactin changes the distribution of GluR2/3 in MIN6 cells. (A) Confocal images of GluR2/3 and cortactin in MIN6 cells. Co-localization of cortactin (left panel) and GluR2/3 (middle panel) was visualized by double immunofluorescence. GluR2/3 was partially co-localized with cortactin. Scale bar, 10 μm. (B) Western blot showing suppression of cortactin expression by RNAi in MIN6 cells. Cell lysates (3 μg per lane) were analyzed. Treatment with two different oligo sequences of siRNA against mouse cortactin suppressed cortactin expression. (C) Intracellular GluR2/3 distribution was changed in cortactin-depleted MIN6 cells. Cortactin and GluR2/3 expression was visualized by double immunofluorescence. Line-scanning of fluorescence intensity for GluR2/3 is shown by the yellow dashed line in two different cells from control (left panels) or cortactin-depleted (right panels) cells. Note that immunoreactivity for GluR2 is more evident around the cell periphery in cortactin-depleted cells. Arrows show cell edges. Scale bar, 20 μm.

Next, we investigated whether the localization of GluR2/3 was modulated by the expression of cortactin in MIN6 cells. Double-immunofluorescence experiments showed that cortactin was visible as dots that were distributed throughout the cell (Fig. 5A). GluR2/3 co-localized with cortactin (36.1±1.6% in total amount of GluR2/3, n=20 cells). We next examined whether cortactin depletion could alter GluR2/3 distribution. The expression of cortactin was suppressed by cortactin-specific RNAi, as measured by Western blotting (Fig. 5B). In control cells, puncta for GluR2/3 were visible through the cells (Fig. 5C left panels). On the other hand, cortactin-depleted cells showed clear dot-like immunoreactivity for GluR2/3, which was often observed at the cell periphery (Fig. 5C right panels). Thus, the expression of cortactin might modulate the localization of GluR2/3 in MIN6 cells.

## Discussion

We demonstrated that expression of GluR2 and GluR3 at protein level in MIN6 cells, a mouse pancreatic β-cell line. And we showed the AMPA-induced internalization of GluR2/3 in MIN6 cells. In addition, an interaction between cortactin and GluR2/3 is important for the maintenance of intracellular localization of GluR2/3; disruption of this interaction alters GluR2/3 distribution. From these results, we propose that like neuronal cells, β-cells have an adaptable mechanism involving GluR2/3 for recognizing and transmitting glutamatergic signals.

In pancreatic islets, α-cells co-release glucagon and glutamate under low-glucose concentrations (Yamada *et al.*, 2001; Hayashi *et al.*, 2003). Released glutamate can activate AMPA receptor in β-cells to stimulate insulin secretion (Bertrand *et al.*, 1992; Wu *et al.*, 2012). Na^+^-dependent glutamate transporters take-up excess glutamate from the extracellular space (Weaver *et al.*, 1998). Langerhans islets have glutamatergic signaling capabilities (such as glutamate output, input, and signal termination) (Takahashi *et al.*, 2019). While these findings implicate a role for AMPA receptors in insulin secretion from β-cells, the signal recognition mechanisms that occur via glutamate receptors are not fully understood. In this study, we found that AMPA receptor containing GluR2 was internalized upon AMPA stimulation under low-glucose conditions. To our knowledge, this is the first demonstration that endogenous AMPA receptor internalization occurs in non-neuronal cells. In addition, the internalization of GluR2 in MIN6 cells is very similar to that in neuronal cells. GluR2/3 was present not only at the cell surface but also intracellularly, and was often co-localized with the clathrin-pit marker proteins AP2 and CHC (Fig. 1 and Fig. 2). These results suggest that GluR2/3 in MIN6 cells undergoes clathrin-mediated endocytosis, as is the case in neuronal cells (Carroll *et al.*, 1999; Man *et al.*, 2000). After stimulation of MIN6 cells with AMPA, internalized GluR2 largely localized in endosomes (Fig. 3). It is possible that GluR2 transported to recycling pathway from endosomes back to the plasma membrane.

Pancreatic β cells is known to induce cell depolarization, calcium entry and actin remodeling in response to high glucose (Arous and Halban, 2015). It is conceivable that high glucose-induced cellular activity of β-cells can affect GluR2/3-internalization as well as insulin secretion. Interaction between GluR2/3 and cortactin may be important for GluR2/3 intracellular trafficking in β-cells as well as in neuron. In this study, we could not clarify the detail mechanism of GluR2/3 trafficking regulated by cortactin. Cortactin, a F-actin binding protein, also interacts with actin-related proteins including the Arp2/3 complex (Weed *et al.*, 2000), N-WASP (Weaver *et al.*, 2001), and dynamin (Yamada *et al.*, 2013, 2016). Furthermore, cortactin promotes WASP-induced actin polymerization and stabilizes actin branches (Weaver *et al.*, 2001, 2002). These actin remodeling participates in receptor-mediated endocytosis (Schafer, 2002; Cao *et al.*, 2003; Zhu *et al.*, 2005), endosomal/lysosomal maturation (Kirkbride *et al.*, 2012). In the study, a lot of internalized GluR2/3 were observed in MIN6 cells cultured in high glucose medium (Fig. 1, Fig. 2 and Fig. 5). Under the conditions, depletion of cortactin in MIN6 cells altered the distribution of GluR2/3, suggesting that actin remodeling coordinated by cortactin could partially regulate the GluR2/3 trafficking. Currently, it is unclear how cortactin and actin modulates AMPA-type receptor trafficking in both neurons and MIN6 cells; further studies are required to ascertain this.

As MIN6 cells are neuroendocrine cells that share several properties of neurons, including expression of neurotransmitter receptors, exocytic, endocytic proteins, and so on (Satin and Kinard, 1998). In pancreatic islets, the intercellular signaling using neurotransmitters implicates hormonal regulation, which could be also affected by released hormons and blood glucose (Takahashi *et al.*, 2019). The difference may give a clue to assist the analysis of the molecular mechanism that underlies AMPA receptor internalization in pancreatic β-cells. Our study provides a first step toward understanding the receptive mechanisms, which involve AMPA receptors, used by β-cells to detect and respond to glutamatergic signaling.

## Funding

This work was supported in part by grants from the Ministry of Education, Culture, Sports, Science and Technology of Japan (Grant no. 19H03225 to KT and Grant no. 20K08591 to HY).

## Availability of data and materials

All data generated or analyzed during this study are included in this published article.

## Authors’ contributions

HY, KT, and TML designed the research and wrote the paper. HY, TA, TML, SS and NK performed immunofluorescence, pull down, and immunoprecipitation experiments. SL and MW performed immunoelectron microscopy. TML and KF analyzed data. All authors read and approved the final manuscript.

## Ethics approval and consent to participate

Not applicable.

## Patient consent for publication

Not applicable.

## Competing interests

The authors declare that they have no competing interests.
